# Emerging Cell-Based Therapies in Chronic Lung Diseases: What About Asthma?

**DOI:** 10.3389/fphar.2021.648506

**Published:** 2021-04-20

**Authors:** Andressa Daronco Cereta, Vinícius Rosa Oliveira, Ivan Peres Costa, João Pedro Ribeiro Afonso, Adriano Luís Fonseca, Alan Robson Trigueiro de Souza, Guilherme Augusto Moreira Silva, Diego A. C. P. G. Mello, Luis Vicente Franco de Oliveira, Renata Kelly da Palma

**Affiliations:** ^1^ Department of Surgery, School of Veterinary Medicine and Animal Sciences, University of São Paulo, São Paulo, Brazil; ^2^ Department of Physical Therapy, EUSES University School, University of Barcelona/University of Girona (UB-UdG), Barcelona, Spain; ^3^ Research Group on Methodology, Methods, Models, and Outcomes of Health and Social Sciences (M_3_O), University of Vic - Central University of Catalonia, Vic, Spain; ^4^ Department of Master’s and and Doctoral Programs in Rehabilitation Sciences, Nove de Julho University, São Paulo, Brazil; ^5^ Department of Experimental Cardiorrespiratory Physiology, Postgraduate Program in Human Movement and Rehabilitation, School of Medicine, University Center of Anápolis (UniEVANGELICA), Anápolis, Brazil; ^6^ Institute for Bioengineering of Catalonia, Barcelona, Spain

**Keywords:** chronic lung diseases, asthma treatments, cell-based therapies, mesenchymal stromal cells, extracellular vesicles, immune cells

## Abstract

Asthma is a widespread disease characterized by chronic airway inflammation. It causes substantial disability, impaired quality of life, and avoidable deaths around the world. The main treatment for asthmatic patients is the administration of corticosteroids, which improves the quality of life; however, prolonged use of corticosteroids interferes with extracellular matrix elements. Therefore, cell-based therapies are emerging as a novel therapeutic contribution to tissue regeneration for lung diseases. This study aimed to summarize the advancements in cell therapy involving mesenchymal stromal cells, extracellular vesicles, and immune cells such as T-cells in asthma. Our findings provide evidence that the use of mesenchymal stem cells, their derivatives, and immune cells such as T-cells are an initial milestone to understand how emergent cell-based therapies are effective to face the challenges in the development, progression, and management of asthma, thus improving the quality of life.

## Introduction

Chronic inflammation can affect the respiratory system in many ways, causing damage to the lungs and airways. Asthma is the most common condition that affects the airways, contributing to high absenteeism at work and school. It is the major respiratory disease affecting children (Guibas et al., 2015). Without a known cause, asthma has a multifactorial background and many risk factors associated with its development. Most of the risk factors are related to early life events, such as genetics, several pathogens, and environmental exposures like tobacco smoke and air pollution (Toskala and Kennedy, 2015; World Health Organisation, 2018; Tiotiu et al., 2020). Asthma is an inflammatory and heterogeneous chronic disease. It causes hyperresponsiveness of the airways and exacerbated mucous secretion. It is characterized by recurrent events of cough, wheezing, shortness of breath, and chest tightness, which persistently lead to airflow limitation. (Toskala and Kennedy, 2015; Papi et al., 2018; Mattiuzi and Lippi, 2020). Asthma prevalence has been increasing over the past 50 years affecting around 340 million people worldwide, both adults and children (Mattiuzi and Lippi, 2020). In low and lower-middle-income countries, it accounts for most asthma-related deaths (World Health Organisation, 2018). The mortality rates of this disease have declined recently. However, there are still some associated comorbidities, such as systemic arterial hypertension and pulmonary hypertension, both of which are related to the number of asthma deaths (Kaplan et al., 2020; Miethe et al., 2020; Branco et al., 2020).

**FIGURE 1:**
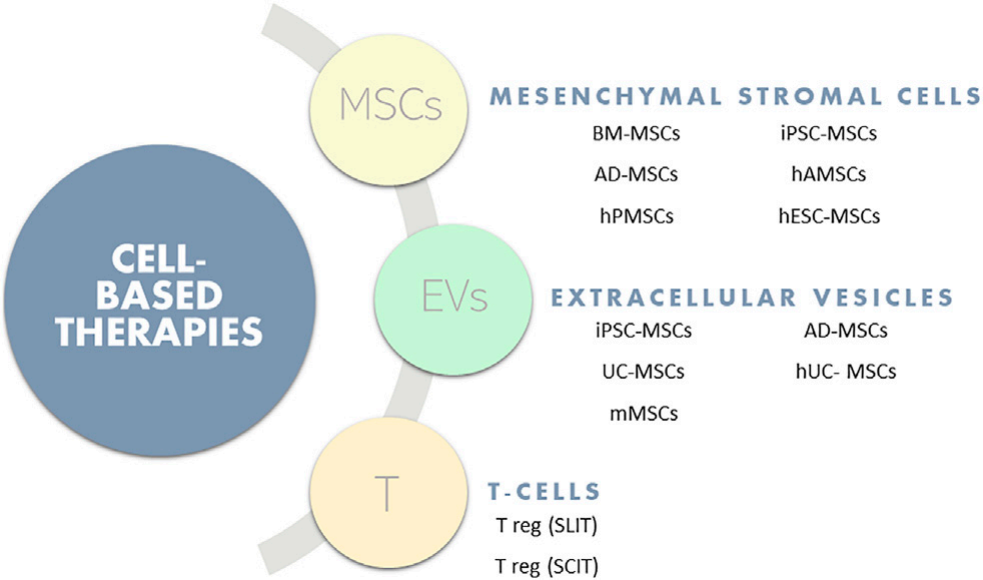
Summary of cell-based therapies for Asthma.

There are many recognized phenotypes designating asthma. However, they can be summarized into i) allergic, in early onset, mild, or moderate-to-severe remodeled asthma or ii) non-allergic with late-onset eosinophilic asthma or non-eosinophilic asthma (Kaur, Chupp, 2019). Moreover, asthma can be categorized by endotypes related to the biomarkers involved in the disease mechanisms (Chung, 2018). The traditional asthma treatment is corticosteroid drugs, which have been used since the middle of the last century (Baldwin et al., 1961). For many patients, inhaled corticosteroids, in combination or not with long-acting β2 agonists, are an effective treatment, suppressing airway inflammation and constriction, although it is not curative. However, 5–10% of asthmatic patients do not respond to steroid-based therapies (Wang et al., 2010). This leads to severe steroid-resistant asthma (SSR), which is associated with non-eosinophilic endotypes of the disease, including neutrophilic asthma (Kim et al., 2017) As demonstrated in Figure 1, some alternatives to the classical treatment of choice for asthma have arisen and most research has focused on cell-based therapies. The most common is the use of mesenchymal stem cells. However, studies on extracellular vesicles and immune modulation by using T-cells are recently increasing. Although these options are mainly in the pre-clinical step, they may be a promising approach, especially in cases of refractory asthma, in which the use of corticosteroids is no longer a viable option.

### Cell-Based Therapies in Asthma

Initial studies of cell-based approaches to repair a damaged lung started two decades ago, using hematopoietic stem cells. Since then, other types of cells have been explored, such as mesenchymal stem cells, endothelial progenitor cells, or even embryonic stem cells (Kim et al., 2017). In this review, we summarized the advancements in cell therapy involving mesenchymal stem cells, extracellular vesicles, and immune cells (T-cells) in pre-clinical and clinical trial studies (Table 1).

**TABLE 1:** Cell-based therapies in Asthma.

Author	Study type		Route	Therapy	Results
Kitoko et al. (2018)	PC- HDM	IT		BM-MSCs	Did not reduce lung parenchyma inflammation, airway hyperresponsiveness or mucus hypersecretion in asthma model
Habibian, et al. (2018)	PC- OVA	IV		BM-MSCs	Reduced eosinophilia, inhibited expressions of Th2 and Th17 cytokines and elevated levels of regulatory T cells (tregs) cytokines
Abreu et al. (2018)	PC- HDM	IT		BM-MSCs	Modulated macrophages toward an anti-inflammatory phenotype, increased secretion of pro-resolution and anti-inflammatory mediator, consequently reducing the remodeling process in asthma model
Boldrini-Leite et al. (2020)	PC-OVA	IT		BM-MSCs	Decreased eosinophilia, lymphocyte, total protein, IL-13, IL-17 A levels and decreased airway remodeling, which persisted 14 days after injection in asthma model
Cui et al. (2020)	PC- CE	IT		BM-MSCs and macrophages	Polarized macrophages to an anti-inflammatory phenotype (M2), apparently by aryl hydrocarbon receptor (AhR) signaling activation in asthma model
Dai et al. (2017)	PC-OVA	IV		AD-MSCs	Decreased airway responsiveness, infiltrations of inflammatory cells with are associated impaired regulation of Foxp3, IL17, IL10 and RORγ expression and restored the percentage of CD4^+^CD25 + Foxp3+ tregs in the spleen in asthma model
Dai et al. (2018)	PC-OVA	IT		AD-MSCs	The restoration of Th1/Th2 cell balance in asthma model
Halim et al. (2019)	PC-OVA	Aerosol		AD-MSC-pANGPT1	Reduced the expression of pro-inflammatory cytokine genes (IL- 4, TNF, TGF-β, and MMP-9) in asthma model
Hur et al. (2020)	PC-OVA	IV		AD-MSCs or BM-MSCs	Increased Th2 levels and inflammation cell infiltration in asthma model
Li et al. (2017)	PC-OVA	IV		hPMSC	Showed a Th17/Treg rebalance mediated by increased IL-10 levels in asthma model
Li et al. (2018)	PC-OVA	IV		hPMSC	Decreased of notch pathway in asthma model
Dalouchi et al. (2021)	PC-OVA	IV		hAMSC	Decreased in oxidative stress, serum levels of immunoglobulin E (IgE), interleukin 4 (IL-4), transforming growth factor (TGF-β) and increased interferon gamma (IFN-γ) and IL-10 in asthma model
Yao et al. (2018)	PC-OVA	IT		iPSC-MSCs	Connexin 43 regulate TNTs formation by mediating the mitochondria transfer from iPSC-MSCs to epithelial cells. The mitochondria prevented epithelial cells apoptosis reducing the allergic airway inflammation in an asthma model
Zhong et al. (2019)	PC-OVA	IV		iPSC-MSCs	Reduced collagen deposition and airway thickening via regulating the expression of signaling molecules of the TGF-β1/Smad pathway in asthma model
Lin et al. (2018)	PC-OVA	IV		hESC-MSCs	Decreased Th2 and treg cells by 47 protein-coding mRNAs in asthma model
Du et al. (2018)	PC- PBMCs	*In vitro*		Exosomes secreted by BM- MSCs	Tregs modulation in IL-10 and TGF-β1 increase, probably due to antigen presenting cells (APCs) modulation and not CD4^+^ T cells in asthma *in vitro* model
Lin et al. (2019)	PC- PBMCs	*In vitro*		hPMSCs	Reduced the CD4^+^ and CD8+T activation and proliferation and significantly decreased IL-5 levels in asthma *in vitro* model
Aguiar et al. (2020)	CT	IV		BMMCs	No serious adverse events. Lung function remained stable throughout. A slight increase in ventilation of the right lung
Zhuansun et al. (2019)	PC- PBMCs	*In Vitro*		Exosomes secreted by BM- MSCs	miR-1470 which promote the differentiation of CD4^+^CD25 + FOXP3+ tregs in PBMCs of asthmatic patients by inducing the expression of P27KIP1
Cruz et al. (2015)	PC- AHE	IV		EVs by hBM-MSC and mMSCs	EVs from hMSCs demonstrated be more potent than those from mMSCs in asthma model
de Castro et al. (2017)	PC-OVA	IV		EVs by AD-MSC	EVs acting different from MSCs regarding to lung mechanic, pro-inflammatory mediators, and regulatory T cells in asthma model
Fang et al. (2020b)	PC- OVA	IV		sEV by iPSC-MSCs	Reduced M2 macrophages in an asthma model and suggested that EVs presents 312 proteins that can be involved in the therapeutic effects
Dong et al. (2021)	PC-OVA	IV		EVs by hUC- MSC	Increased of miR-146a-5p acting on inflammation and airway remodeling by decrease pro-inflammatory mediators (IL-13and IL-4) and the expression of pro fibrogenic markers (α-SMA, collagen-1, and TGF-β1-*p*-smad2/3 signaling pathway in asthma model
Hoshino et al. (2019)	CT	SLIT		HDM tablet	Reduced FeNO (fractional exhaled nitric oxide), eosinophilic airway inflammation and improved the airflow limitation in asthmatic patients
Xian et al. (2020)	CT	SLIT and SCIT		Der-p and der-f	SLIT and SCIT upregulated CD4^+^CD25 + FoxP3+ tregs, however only SLIT showed clinical improvement related to tregs in asthmatic patients
Skuljec et al. (2017)	PC- OVA	IV		CAR tregs	CAR tregs reduced airway hyper-reactivity, eosinophilic airway inflammation and cell infiltrates in the lung. Moreover, prevented excessive mucus production and increased allergen-specific IgE and Th2 cytokine levels

BM-MSC, bone marrow-derived mesenchymal stromal cells; AD-MSCs, adipose-derived mesenchymal stem cells; hAMSC, human amniotic membrane; IT, intratracheal; IV, intravenous; hPMSCs, placental-derived mesenchymal stromal cells; iPSC-MSCs human induced pluripotent stem cell; hESC-MSCs, human embryonic stem cells; PBMCs, peripheral blood mononuclear cells; EVs, Extracellular vesicles; sEV, small extrecallular vesicles; UC-MSC, umbilical cord mesenchymal stromal cells; PC, Pre-clinical; CT, Clinical trial; HDM, house dust mite; OVA, ovalbumine; CE, cockroach extract; AHE, Aspergillus fumigatus hyphal extract; SLIT, sublingual immunotherapy; SCIT subcutaneous immunotherapy; Der-p, Dermatophagoides pteronyssinus; Der-f, Dermatophagoides farina.

### Mesenchymal Stromal Cells

Because of reparative and immunological properties, such as the potential to attenuate allergic immune diseases safely, mesenchymal stem cells (MSCs) are being considered as a good approach to treat asthma (Weiss, 2018). MSCs are multipotent, nonhematopoietic, and found in both adult and neonatal tissues. They have different sources, such as bone marrow, adipose tissue, placenta, umbilical cord, and organs such as the lungs (Sueblinvong et al., 2008). However, each source of MSCs can present different anti-inflammatory or regenerative effects (Abreu et al., 2017). This results in various potential mechanisms of the action of MSCs from different sources in asthma. Even with a limited number, the bone marrow-derived mesenchymal stem cells (BM-MSCs) have been commonly used and are well characterized in experimental asthma models (Lathrop et al., 2014; Cruz et al., 2015). Habibian and colleagues (2018) demonstrated that injection of BM-MSCs into the vein reduced eosinophilia and inhibited expressions of Th2 and Th17 cytokines. In addition, it elevated levels of regulatory T cells (Tregs) cytokines in an experimental model of allergic asthma induced by ovalbumin (OVA). On the other hand, the intratracheal (IT) route delivers cells directly to affected airways. The BM-MSCs IT injection in OVA-induced asthma decreased eosinophilia, lymphocyte, total protein, IL-13, and IL-17A levels and significantly decreased airway remodeling, which persisted for 14 days after the injection (Boldrini-Leite et al., 2020).

OVA-induced asthma is widely used as an asthma model. However, it is not clinically relevant to humans compared with allergen and grass pollen. Therefore, allergens such as house dust mite (HDM) have been considered more clinically relevant, exhibiting inflammatory and ultrastructural changes in lung parenchyma and the airways, similar to human disease (Kitoko et al., 2018). However, the IT injection of BM-MSCs cannot reduce lung parenchyma inflammation, airway hyperresponsiveness, or mucus hypersecretion in HDM-induced asthma (Kitoko et al., 2018). Considering this limitation, a recent study demonstrated that a pre-treatment with eicosapentaenoic acid (EPA) could potentiate the BM-MSC-based therapy in HDM-induced asthma. This leads to the modulation of macrophages toward an anti-inflammatory phenotype, increased secretion of pro-resolution and anti-inflammatory mediators, consequently reducing the remodeling process (Abreu et al., 2018). The cockroach extract (CE) is another allergen that can lead to asthma and increase asthma morbidity in children (Pomés et al., 2019). In a murine cockroach allergen (CRE)-induced asthma model, the injection of BM-MSC modulate macrophage differentiation from a pro-inflammatory phenotype (M1) to an anti-inflammatory phenotype (M2), apparently by aryl hydrocarbon receptor (AhR) signaling activation. The AhR is a ligand-activated receptor that mediates the toxicity of environmental pollutants and can induce molecular cascade mediated by BM-MSC immunosuppression. Future studies should be addressed to understand the link between MSC and AhR during immunomodulation activity in asthma (Cui et al., 2020).

Due to the high immunomodulatory capacity in BM-MSC, adipose-derived mesenchymal stem cells (ADMSCs) could have therapeutic potential in asthma. ADMSCs injected into the vein of a mouse with OVA-induced asthma decreased airway responsiveness, infiltrations of inflammatory cells that are associated with impaired regulation of Foxp3, interleukin (IL)17, IL10, and RORγ expression and restored the percentage of CD4^+^CD25 + Foxp3+ Tregs in the spleen (Dai et al., 2017). The restoration of Th1/Th2 cell balance mediated by ADMSCs may be the mechanism associated with airway responsiveness and inflammation decrease in OVA-induced asthma (Dai et al., 2018). However, ADMSCs can be enhanced by gene transfection. The MSC-pANGPT1 (angiopoietin 1 gene) were aerosolized in a rabbit OVA-induced asthma and reduced the expression of pro-inflammatory cytokine genes (IL-4, TNF, TGF-β, and MMP-9), which can be an additional beneficial effect in asthma treatment (Halim et al., 2019). Apart from cell source, the cell dose, cell injection frequency, and injection site are crucial in MSC therapy. Hur and colleagues (2020) showed that double injection of ADMSCs and BM-MSCs in OVA-induced asthma increased Th2 levels and inflammatory cell infiltration. Therefore, both ADMSCs and BM-MSCs can be used for asthma treatment. However, the frequency of injection should be used carefully.

MSCs derived from adult tissues can present limited therapeutic benefits due to their cell variability quality from different donors and their limitation in proliferative capacities (Crisostomo et al., 2006). Hence, human placenta MSCs (hPMSCs) could be an alternative source of stem cells for therapeutic use. The hPMSCs have few ethical issues, are easily obtained, and demonstrate prominent inhibitory effects on T cells compared with BM-MSCs (Li et al., 2014). The hPMSC, from the placental tissue of a healthy pregnant mother, was injected into a rat OVA-induced asthma for the first time in 2017. It showed a Th17/Treg rebalance mediated by increased IL-10 levels (Li et al., 2017). A further study suggested that the Notch pathway, associated with the induction of Th2 in asthma, decreased its expression after treatment with hPMSC (Li et al., 2018). Moreover, a recent study demonstrated that the human amniotic membrane (hAM) could be an ideal mesenchymal source. This is because it can decrease oxidative stress, serum levels of immunoglobulin E (IgE), IL4, transforming growth factor (TGF-β), and increase interferon-gamma (IFN-γ) and IL-10 in the OVA-induced asthma model (Dalouchi et al., 2021).

It is well known that human-induced pluripotent stem cells (iPSC-MSCs) have emerged as another valuable mesenchymal source compared with MSCs derived from adult tissue due to their higher proliferative and differentiation capacity, longer life span, and more substantial immune privilege (Gao et al., 2017). A study using an OVA-induced asthma model showed that connexin 43 regulates TNTs formation by mediating the mitochondria transfer from iPSC-MSCs to epithelial cells. The mitochondria prevent epithelial cell apoptosis, reducing the allergic airway inflammation in an asthma model (Yao et al., 2018). Furthermore, iPSC-MSC administered in a chronic asthma model can reduce collagen deposition and airway thickening via regulating the expression of signaling molecules of the TGF-β1/Smad pathway (Zhong et al., 2019). This shows a potential therapeutic effect in the treatment of acute or chronic asthma. However, iPSCs present genetic instability and tumorigenicity after reprogramming *in vivo* (Tan et al., 2014). Nonetheless, this requires further studies before their use in clinical applications. On the other hand, MSCs derived from human embryonic stem cells (hESC-MSCs) present the same advantages as iPSC-MSC with less risk of genetic instability. This may decrease Th2 and Treg cells by 47 protein-coding mRNAs, which are potential targets for asthma treatment in a future clinical study (Lin et al., 2018).

According to previous demonstrations, the use of MSCs to treat asthma has been successful in different animal models. However, there are still some issues that must be addressed before translating this therapy to humans. To the best of our knowledge, there is only one study (Aguiar et al., 2020) that demonstrated the potential efficacy of MSCs in patients with asthma and one ongoing clinical trial (NCT03137199). However, these studies (Aguiar et al., 2020) have limitations, such as a small sample size with no placebo-control and inflammatory biomarkers. Procedures for cell culture need to be appropriately standardized to produce a satisfactory number of cells and should be proliferative and maintain their regeneration properties. Also, the source of the MSCs and their effects must be accurately known as they may vary according to different types of MSCs (Yu et al., 2020). Therefore, the major challenge is to define efficient protocols to harvest, culture, and deliver the MSCs to obtain a proper therapy. Likewise, effort must be applied in future studies to finally address MSCs in human asthma treatment.

### Extracellular Vesicles

Extracellular vesicles (EVs) are secreted by many types of cells, including MSCs. They circulate in the extracellular space and include exosomes, microvesicles, and apoptotic bodies. These vesicles are implicated in cell-cell communication and are considered essential for homeostasis. EVs usually contain intracellular proteins, lipids, and nucleic acids, as well as miRNAs, cytokines or chemokines, tissue factors, or caspases (Bartel et al., 2020; Nagano et al., 2019; Andres et al., 2020; Cañas et al., 2019; van den Berge and Tasena, 2019). Therefore, the biological factors secreted by MSCs can be the key mechanism in asthma treatment. Furthermore, MSC-EVs are better defined, less complicated, and easier to store compared with MSCs (Konala et al., 2016). The first experiments with EVs in asthma models demonstrated that EVs acted differently from MSCs regarding lung mechanics, pro-inflammatory mediators, and regulatory T-cells (de Castro et al., 2017). Moreover, EVs from hMSCs were more potent than those from mMSCs (Cruz et al., 2015). Therefore, their use must be encouraged with further investigation.

Recent data have shown that intravenous treatment in an experimental model of asthma with EVs derived from iPSC-MSCs can prevent the increase of group 2 innate lymphoid cells (ILC2s), which is responsible for the initiation and maintenance of type 2 allergic airway inflammation, probably through miR-146a-5p. In addition, a protocol of anion-exchange chromatography for isolation of MSC-EVs was standardized in this study. This study demonstrated some advantages when compared with the ultracentrifugation protocol due to the scalable approach and feasibility of application in industrial production (Fang et al., 2020a). Based on this protocol for isolation of MSC-EVs, the same group showed for the first time that this approach reduced M2 macrophages in an asthma model and suggested that EVs present 312 proteins that can be involved in therapeutic effects (Fang et al., 2020b). Conversely, considering that miR-146a-5p is abundant in human umbilical cord MSCs (hUCMSC-EVs) (Song et al., 2017), these EVs could be a potential choice for asthma treatment. Dong and colleagues (2021) demonstrated for the first time that a hypoxia environment for EVs extraction from hUCMSCs can increase the miR-146a-5p level even more in mice with OVA-induced asthma. The increase of miR-146a-5p acts on inflammation and airway remodeling by decreasing pro-inflammatory mediators (IL-13and IL-4) and the expression of profibrogenic markers (α-SMA, collagen-1, and TGF-β1-p-smad2/3 signaling pathway) in OVA mice. Therefore, oxygen depletion can play an important role during the EV secretion. It should, therefore, be considered during asthma treatment with EVs.

On the other hand, Exosomes derived from MSCs presents similar immunomodulatory effect as MSCs. The peripheral blood mononuclear cells (PBMCs) of patients with asthma have mainly been used associated with exosomes in *in vitro* studies. One study where exosomes secreted by BM-MSCs were isolated and cultured with PBMCs from patients with asthma demonstrated Tregs modulation in IL-10 and TGF-β1 increase. This was probably due to antigen-presenting cell (APC) modulation and not CD4^+^ T cells. Thus, BM-MSC exosomes may prevent the disadvantages of BM-MSCs and be a potential therapeutic agent for asthma (Du et al., 2018). Moreover, BM-MSC exosomes presents high level miR-1470 which promote the differentiation of CD4^+^CD25 + FOXP3+ Tregs in PBMCs of asthmatic patients by inducing the expression of P27KIP1(Zhuansun et al., 2019) In another recent study, hPMSCs were cultured with PBMCs from children with asthma, reducing CD4^+^ and CD8+T activation and proliferation and significantly decreasing IL-5 levels (Lin et al., 2019). Therefore, considering the inadequate response to conventional treatment, administration of hPMSCs could be a good option.

After all, it seems that EVs secreted by MSCs are a promising cell therapy for asthma. However, these results should be interpreted with caution because of the limited number of studies. Thus, more evidence is needed.

### Immune Cell Treatment: T-Cells

In response to an asthma attack, there is an elevation in the number of inflammatory cells, activated eosinophils, and T cells. The T cells are responsible for the recognition and reaction against specific antigens, which stimulate the differentiation of naive CD4^+^ T cells into Th2 cells and Tregs. These Tregs suppress an excessive immunological response of Th2. However, when the Tregs are dysfunctional and cannot control Th2, asthma can develop (Kawayama et al., 2018). In this sense, treatment based on Tregs by increasing their number and enhancing their suppression function could be a good alternative for asthmatic patients.

Antigen-specific immunotherapy (ASIT) is the most used method for Tregs induction, and it can be administrated by sublingual immunotherapy (SLIT) (Hoshino et al., 2019) and subcutaneous immunotherapy (SCIT) (Lozano et al., 2014). There are promising clinical trials regarding the use of SCIT in asthmatic patients and HDM (house dust mite), where SLIT tablets are often used. Hoshino et al., 2019 showed that HDM SLIT tablet reduced fractional exhaled nitric oxide (FeNO), eosinophilic airway inflammation, and improved the airflow limitation in patients with asthma. On the other hand, the administration SLIT and SCIT for Dermatophagoides pteronyssinus (Der-p) and Dermatophagoides farinae (Der-f), the most common allergens in patients with asthma in China, demonstrated similar efficacy in an asthmatic patient, mediated from different mechanisms. SLIT and SCIT upregulated CD4^+^CD25 + FoxP3+ Tregs. However, only SLIT showed clinical improvement related to Tregs (Xian et al., 2020). Despite these results, SCIT is just recommended as an adjunct to pharmacotherapy in individuals with asthma, and SLIT is not recommended (Cloutier et al., 2020).

On the other hand, Tregs can be engineered *ex vivo* with a chimeric antigen receptor (CAR) (Hombach et al., 2009) and provide more specificity regarding to diversity of asthma antigens. Skuljec and colleagues (2017) showed that injection of CAR Tregs in a mouse OVA-induced asthma was effective in reduced Th2, mucus hypersecretion, airway hyper-reactivity and allergen-specific IgE.Further studies should be addressed to identify asthma triggers and improve treatments based on Treg.

## Conclusion

Animal models and clinical trials suggest that cell-based therapies are a potential strategy for asthma treatment, although these therapies are still on preliminary studies or the pre-clinical testing phase. The use of mesenchymal stem cells to treat asthma is still a major discussion because it lacks translation to humans. To date, most of the evidence relies on animal studies and only a few on human subjects, including an ongoing clinical trial.

Another possibility is the use of extracellular vesicles or T-cell. However, current studies still lack profound knowledge on the effects of EVs. In conclusion, the effects of these cells and derivatives can be addressed to reduce responsiveness and inflammation according to preliminary studies. This can contribute to improving lung function, immunity response, and tissue regeneration/repair at the bronchial level. Although research on these topics is still at the initial stage, more evidence and research efforts are warranted.

The use of mesenchymal stem cells, their derivatives, and immune cells such as T-cells are just the initial milestones to understand how emergent cell-based therapies are effective to face the challenges of the development, progression, and management of asthma, thus improving the quality of life.
